# The Prescriber

**Published:** 1887-03

**Authors:** 


The Prescriber. A Dictionary of the New Therapeutics. By John H. Clarke, M. D., Edin., London: Keene & Ashwell, 1886.

This is the second edition of this neat little book. The first edition was duly reviewed in the pages of this Journal in January, 18 6, page 45.
W. M. J.

